# Supplementary material for: Simulation of miniscrew-root distance available for molar distalization depending on the miniscrew insertion angle and vertical facial type
Source: PLoS One. 2020 Sep 24;15(9):e0239759. doi: 10.1371/journal.pone.0239759 (PMC7514046; doi:10.1371/journal.pone.0239759)
Supplement: Attachment — Submitted filename: Responses to the Reviewers.docx [file pone.0239759.s002.docx]

**Responses to the Reviewers’ Comments**
Manuscript number: PONE-D-20-04649R1

Title: Simulation of miniscrew-root distance for possible molar distalization depending on miniscrew insertion angle and vertical facial types

**Reviewer #1:**

Reviewers' requests and comments have been successfully addressed and the manuscript was improved.
I have some remaining minor points:

**Comment 1:** TITLE
A suggestion for the title: “Simulation of miniscrew-root distance available for molar distalization depending on miniscrew insertion angle and vertical facial types.”

**Response:**

Thank you for your helpful suggestion. We have now revised the title according to your suggestion, as well as the corresponding parts in the revised manuscript. We appreciate your comment, as we believe your input has improved our submission.

**Revision:**

The title was revised.

Before:

“Simulation of miniscrew-root distance for possible molar distalization depending on miniscrew insertion angle and vertical facial types.”

After:

“Simulation of miniscrew-root distance available for molar distalization depending on the miniscrew insertion angle and vertical facial type.”

**Comment 2:** MATERIAL AND METHODS
Please provide the reference studies citation following the sentence referring to study sample size (page 6 lines 144-145).

**Response:**

Thank you for your comment and we apologize for this issue. As requested, we have now added the citations for the reference studies.

(page 6, lines 141-143)

Since our study is an explorative pilot study, 20 subjects for each group were determined in consideration of sample numbers suggested as suitable for the pilot study [19-21].

(page 24, lines 509-514)

References

19.	Julious SA. Sample size of 12 per group rule of thumb for a pilot study. Pharmaceutical Statistics: The Journal of Applied Statistics in the Pharmaceutical Industry. 2005;4(4):287-91.

20.	Isaac S, MICHAEL W. Handbook in Research and Evaluation. San Diego, Californ4ia: ED. ITS Publishers; 1971.

21.	Van Belle G. Sample size. Wiley Hoboken (NJ); 2008. p. 27-52.

**Comment 3:** DISCUSSION
As a suggestion, the following sentence should be removed: “Taking this into consideration, when measuring the miniscrew-root distance in this study, the distance from the lamina dura to the miniscrew surface can be measured.”, since it is reported in the following sentence that the lamina dura is a difficult structure to be measured in CBCTs (page 21, lines 428-429).

**Response:**

We appreciate your insightful comment. We agree with your suggestion and have now removed the sentence in the revised manuscript.

**Comment 4:** CONCLUSION
Please check the sentence: “In the Mx 5-6, Mn 5-6, and Mn 6-7 regions, but not in the Mx 6-7 region, an increase in the miniscrew insertion angle was found to significantly increase the miniscrew–root distance.” (page 22, lines 459-461) According to the results of GEE analysis presented in Table 6, the miniscrew–root distance increased as the miniscrew placement angle increased (p<.001). This result doesn’t mention any exception, so the following statement should be checked: “but not in the Mx 6-7 region” and “except in the Mx 6-7 region” in the conclusions of the manuscript and the abstract, respectively.

**Response:**

We would like to thank you for your suggestion, and we agree with your comments. In the abstract and conclusions of the manuscript, we aimed to describe that the pattern of increasing miniscrew-root distance as the miniscrew placement angle increases varies according to the miniscrew placement site and facial type (Table 3, Figure 6, GEE results in Table 6). However, as you have commented, the sentences were unclear and contradictory. The phrases “but not in the Mx 6-7 region” and “except in the Mx 6-7 region” have now been deleted from the conclusions of the manuscript and the abstract, respectively, and the remaining statements have been revised.

**Revision:**

The abstract was revised.

Before:

Miniscrew–root distance increased as the insertion angle increased from 0° to 60° at all placement sites, except in the Mx 6-7 region.

After:

Miniscrew–root distance significantly increased as the insertion angle increased from 0° to 60°.

(page 2, lines 42-43)

Before:

Miniscrew–root distance increased with the increased insertion angle in all posterior interradicular areas, except in the Mx 6-7 region and in the hypodivergent facial type.

After:

Miniscrew–root distance increased significantly with the increased insertion angle, and the amount of increase was affected by the miniscrew placement site and vertical facial type.

(page 2, lines 49-50; page 3, line 51)

The conclusion was revised.

Before:

In the Mx 5-6, Mn 5-6, and Mn 6-7 regions, but not in the Mx 6-7 region, an increase in the miniscrew insertion angle was found to significantly increase the miniscrew–root distance.

After:

An increase in the miniscrew insertion angle was found to significantly increase the miniscrew–root distance, and the amount of increase was affected by the miniscrew placement site and vertical facial type.

(page 22, lines 452-455)

**Reviewer #2:**

The authors have satisfactorily addressed all my concerns and therefore I recommend publication of this manuscript.
